# Post-transfer reassessment for endovascular treatment: factors associated with changes in EVT eligibility

**DOI:** 10.1186/s42466-026-00539-8

**Published:** 2026-10-09

**Authors:** Daniel P.O. Kaiser, Julia Holzer, Daniela Schoene, Volker Puetz, Matthias Gawlitza

**Affiliations:** 1 Institute and Polyclinic for Diagnostic and Interventional Neuroradiology, Faculty of Medicine, University Hospital Carl Gustav Carus, TUD Dresden University of Technology, Dresden, Germany; 2 Dresden Neurovascular Center, University Hospital Carl Gustav Carus, TUD Dresden University of Technology, Dresden, Germany; 3 Department of Neurology, Faculty of Medicine, University Hospital Carl Gustav Carus, TUD Dresden University of Technology, Dresden, Germany; 4 Section of Neuroradiology, Jena University Hospital, Jena, Germany

**Keywords:** Acute ischemic stroke, Endovascular treatment, Interhospital transfer, Large vessel occlusion, Repeat imaging, CT angiography, ASPECTS

## Abstract

**Background:**

Repeat imaging after interhospital transfer for endovascular treatment (EVT) remains controversial because EVT eligibility may change during transfer, whereas routine repeat imaging may delay reperfusion. We investigated factors associated with complete recanalization and extensive infarct progression, and their implications for post-transfer reassessment.

**Methods:**

We retrospectively analyzed 200 consecutive patients with anterior circulation large vessel occlusion transferred for EVT between 2016 and 2020 who underwent repeat non-contrast CT, CT angiography, and serial neurological assessment. Primary outcomes were complete recanalization on repeat CT angiography and extensive infarct progression (ASPECTS 0–3) precluding EVT. Factors associated with both outcomes were evaluated using multivariable Firth penalized logistic regression.

**Results:**

Changes affecting EVT eligibility occurred in 19 patients (9.5%), including complete recanalization in 10 (5.0%) and extensive infarct progression in 9 (4.5%). Intravenous thrombolysis was associated with complete recanalization (OR 4.87, 95% CI 1.08–46.25; *P* = 0.039), while greater neurological improvement showed a consistent direction of association (OR 1.09, 95% CI 1.00–1.18; *P* = 0.059). Greater neurological deterioration (OR 0.83, 95% CI 0.70–0.97; *P* = 0.018) and lower baseline CTA source-image ASPECTS (OR 0.55, 95% CI 0.34–0.79; *P* = 0.001) were associated with extensive infarct progression.

**Conclusions:**

Approximately one in ten transferred patients experienced clinically meaningful changes affecting EVT eligibility. Neurological reassessment combined with baseline CTA source-image ASPECTS may help identify patients most likely to benefit from repeat imaging and supports a selective rather than routine repeat imaging strategy after interhospital transfer.

**Supplementary Information:**

The online version contains supplementary material available at https://doi.org/10.1186/s42466-026-00539-8.

## Introduction

Endovascular treatment (EVT) is the standard of care for acute ischemic stroke caused by anterior circulation large vessel occlusion (LVO) in eligible patients [1]. Because the benefit of reperfusion declines rapidly with time, minimizing treatment delays throughout the acute stroke pathway remains a central objective of modern stroke care [2, 3]. 

Many patients initially present to primary stroke centers (PSC) and subsequently require transfer to comprehensive stroke centers (CSC) for EVT [4, 5]. During interhospital transfer, however, cerebral perfusion and neurological status may evolve substantially. Spontaneous or thrombolysis-associated recanalization may eliminate the need for thrombectomy, whereas progressive infarction may reduce the expected benefit of EVT or render patients ineligible for treatment. Consequently, many CSCs routinely perform repeat neuroimaging after transfer. Although repeat imaging may improve patient selection, it delays reperfusion in patients who remain appropriate candidates for EVT. Previous studies have shown that repeat imaging alters treatment decisions in a subset of transferred patients, but they have largely focused on its diagnostic yield or workflow implications rather than identifying which patients are most likely to benefit from reassessment [6]. Consequently, post-transfer imaging strategies continue to vary considerably between stroke centers and are largely determined by local practice rather than evidence-based patient selection.

This question has become increasingly relevant as acute stroke care continues to evolve. Recent guideline updates have expanded EVT eligibility to selected patients with large infarct core, further emphasizing the need to balance rapid treatment with appropriate patient selection [1, 7]. In parallel, the increasing adoption of tenecteplase may increase early recanalization before thrombectomy [8], whereas advances such as flat-panel CT performed in the angiography suite may facilitate rapid exclusion of clinically relevant hemorrhage or extensive infarction in selected patients [9]. Together, these developments highlight the need for a selective approach to post-transfer reassessment rather than a uniform repeat imaging strategy.

We therefore investigated the frequency of clinically meaningful changes during interhospital transfer and sought to identify readily available clinical and imaging factors associated with complete recanalization and extensive infarct progression in patients with anterior circulation LVO referred for EVT.

## Methods

This retrospective observational cohort study was conducted at the University Hospital Carl Gustav Carus Dresden, Germany, a tertiary CSC and coordinating center of the Stroke Network of Eastern Saxony and Southern Brandenburg (SOS-NET). Consecutive adult patients with acute ischemic stroke due to anterior circulation LVO who were transferred for EVT between January 2016 and December 2020 were identified from the institutional prospective stroke registry. The study is reported according to the Strengthening the Reporting of Observational Studies in Epidemiology (STROBE) statement.

The study was approved by the Ethics Committee of the Medical Faculty Carl Gustav Carus, Technische Universität Dresden (approval no. EK272072017). The requirement for written informed consent was waived because of the retrospective analysis of prospectively collected pseudonymized data.

Patients were eligible if baseline non-contrast CT and CT angiography (CTA) from the PSC, repeat CT/CTA performed immediately after transfer, and National Institutes of Health Stroke Scale (NIHSS) assessments at both hospitals were available. Patients with posterior circulation stroke, incomplete imaging datasets, or missing NIHSS documentation were excluded.

Baseline demographic characteristics, vascular risk factors, stroke etiology, workflow metrics, and treatment variables were extracted from the prospective stroke registry. Imaging studies obtained at both hospitals were independently reviewed by two board-certified neuroradiologists blinded to the clinical data, study outcomes, and automated image analysis. In parallel, all examinations were analyzed using a commercially available artificial intelligence platform (Brainomix Ltd., Oxford, UK). Occlusion site, Alberta Stroke Program Early CT Score (ASPECTS), CTA source-image (CTASI) ASPECTS, and collateral status were assessed for every examination in the same reading session, by the same readers independently. Collateral status was assessed using the Tan collateral score on baseline single-phase CT angiography, ranging from 0 (absent collateral filling) to 3 (complete collateral filling), as previously described [10]. Disagreements were resolved by consensus with reference to the automated Brainomix analysis. Stroke severity was assessed using the NIHSS score at both hospitals. The NIHSS scoring at the referring hospital was performed by the treating stroke physician - a trained stroke neurologist at PSC with Stroke unit and at PSC without stroke unit, the trained stroke neurologist from the CSC provided a telestroke assessment [11]. All assessors were trained in NIHSS scoring as part of network stroke care; formal certification, however, was not uniformly documented for every assessor across the 2016–2020 study period.

We defined neurological change during transfer as the difference between the baseline NIHSS at the referring hospital and the NIHSS score upon arrival at the comprehensive stroke center, with positive values indicating neurological improvement and negative values indicating deterioration. During the study period, only patients with a non-contrast ASPECTS ≥ 5 were transferred for EVT evaluation, and intravenous alteplase was the standard thrombolytic agent. All patients in the study had received baseline CTA demonstrating anterior circulation LVO at the PSC.

The primary outcomes were (1) complete recanalization on repeated CTA and (2) extensive infarct progression, defined as an ASPECTS of 0–3 on post-transfer non-contrast CT. This threshold was selected in accordance with current guideline recommendations for EVT eligibility in patients with large infarct cores. During the study period (2016–2020), however, patients with an ASPECTS < 5 were only rarely treated with EVT at our institution, reflecting the evidence and clinical practice before the publication of the randomized trials supporting thrombectomy in selected patients with large infarct cores.

Continuous variables are presented as mean (standard deviation) or median (interquartile range), as appropriate, and categorical variables as frequencies and percentages. Between-group comparisons were performed using Student’s t-test or the Mann–Whitney U test for continuous variables and Fisher’s exact test for categorical variables.

Candidate variables were prespecified a priori based on clinical relevance and published evidence. Because both primary outcomes were rare (10 complete recanalization events and 9 extensive infarct progression events), multivariable Firth penalized logistic regression was used to reduce small-sample bias. Separate models were constructed for complete recanalization and extensive infarct progression. Results are reported as odds ratios (ORs) with profile likelihood 95% confidence intervals (CIs). Analyses were restricted to complete cases.

Prespecified sensitivity analyses included conventional multivariable logistic regression and additional adjustment for interhospital transfer duration. Exploratory analyses evaluated occlusion location (carotid/tandem, M1, or M2) using univariable Firth penalized logistic regression. Because of the limited number of outcome events and the small M2 subgroup, occlusion location was not included in the multivariable models.

Model diagnostics included assessment of multicollinearity, influential observations, and apparent model discrimination. All statistical tests were two-sided with *P* < 0.05 considered statistically significant. Analyses were performed using IBM SPSS Statistics (Version 28; IBM Corp., Armonk, NY, USA) and R (Version 4.5.3; R Foundation for Statistical Computing, Vienna, Austria). Given the retrospective observational design, findings should be interpreted as exploratory, particularly those from the exploratory analyses.

## Results

### Study population

**Fig. 1:**
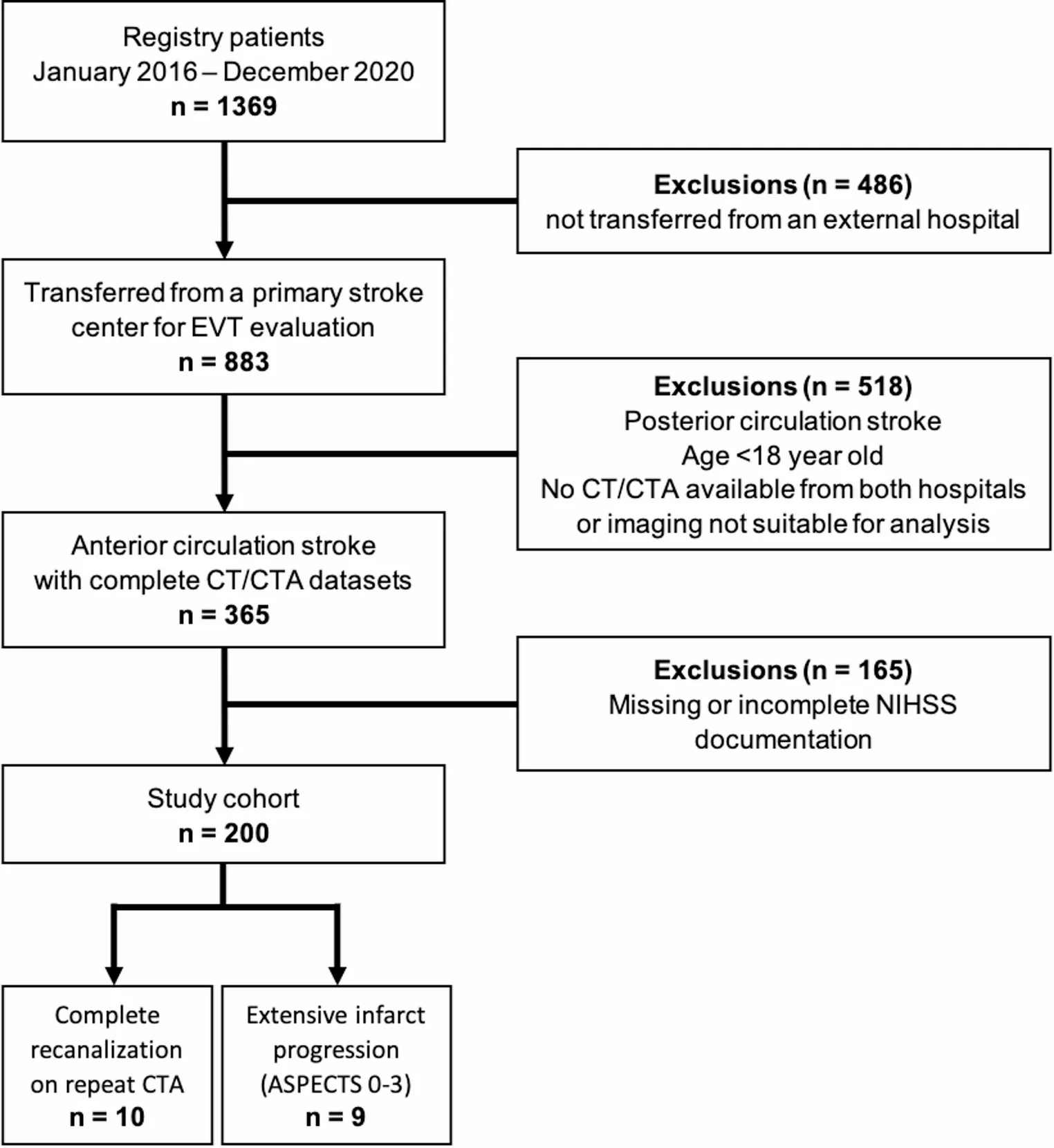
Study flow diagram illustrating patient selection and reasons for exclusion. Among 200 included patients, complete recanalization on repeat CT angiography occurred in 10 patients and extensive infarct progression (ASPECTS 0–3) on repeat non-contrast CT in 9 patients

From 1389 patients who were assessed for EVT eligibility between January 2016 and December 2020, 883 patients were transferred from a primary stroke center. Of these, 200 consecutive patients with anterior circulation LVO fulfilled the inclusion criteria and were included in the analysis (Fig. 1). Baseline characteristics are summarized in Table 1. Clinically meaningful changes on repeated non-contrast CT/CTA affecting EVT eligibility occurred in 19 patients (9.5%), including complete recanalization before angiography in 10 patients (5.0%) and extensive infarct progression (ASPECTS 0–3) on repeat imaging in 9 patients (4.5%).

**Table 1:** Baseline characteristics

Characteristic	Overall (*n* = 200)
**Demographics**	
Age (years)	77 (67–83)
Male sex	88 (44.0)
**Medical history**	
Hypertension	174/196 (88.8)
Diabetes mellitus	82/196 (41.8)
Atrial fibrillation	116/196 (59.2)
Previous stroke	43/196 (21.9)
Dyslipidemia	41/158 (25.9)
Previous myocardial infarction	12/158 (7.6)
Anticoagulation	166 (83.0)
**Stroke characteristics**	
Occlusion site	Supplementary Table 2
Extracranial internal carotid artery stenosis/occlusion (> 90%)	39 (19.5)
**Clinical variables**	
NIHSS at referring hospital	16 (11–19)
Intravenous thrombolysis	112 (56.0)
**Imaging**	
ASPECTS at referring hospital	9 (8–10)
CTA source-image ASPECTS	7 (6–9)
Collateral score	2 (1–2)
**Workflow**	
Onset-to-imaging at referring hospital (min)	81 (61–132)
Onset-to-IVT, (min) *	115 (90–144)
Door-in–door-out time (min)	108 (85–131)
Transfer duration † (min)	148 (119–173)
Door-to-imaging at CSC (min)	12 (9–17)
Onset-to-imaging at CSC (min)	233 (196–316)
**Clinical evolution during transfer**	
NIHSS at comprehensive stroke center	16 (10–20)
Change in NIHSS during transfer ‡	0 (− 3 to 3)
ASPECTS at comprehensive stroke center	8 (6–9)

Data are presented as median (interquartile range) or n (%)

*Among patients receiving intravenous thrombolysis

†Door-out at the referring hospital to arrival at the comprehensive stroke center

‡Positive values indicate neurological improvement during transfer; negative values indicate neurological deterioration during transfer

ASPECTS, Alberta Stroke Program Early CT Score; CTA, computed tomography angiography; CSC, comprehensive stroke center; IVT, intravenous thrombolysis; NIHSS, National Institutes of Health Stroke Scale

**Table 2:** Multivariable Firth penalized logistic regression analyses of factors associated with clinically meaningful changes during interhospital transfer

Variable	OR (95% CI)	*P* value
**Panel A. Complete recanalization**		
NIHSS change during transfer	**1.09 (1.00–1.18)**	0.059
Intravenous thrombolysis	**4.87 (1.08–46.25)**	**0.039**
**Panel B. Extensive infarct progression**		
NIHSS change during transfer	**0.83 (0.70–0.97)**	**0.018**
CTA source-image ASPECTS	**0.55 (0.34–0.79)**	**0.001**
Collateral score	0.33 (0.08–1.14)	0.080

NIHSS change was calculated as the NIHSS score at the referring hospital minus the NIHSS score at the comprehensive stroke center. Positive values indicate neurological improvement and negative values indicate neurological deterioration. Odds ratios are expressed per 1-point increase in NIHSS change

*P* values <0.05 (bold) are considered statistically significant

### Complete recanalization

Baseline characteristics according to recanalization status are presented in Supplementary Table 1 A. Patients with complete recanalization had a high baseline CTASI ASPECTS (median 10 vs. 7; *P* = 0.003) and good collaterals, a near-normal CT ASPECTS (median 10), and marked neurological improvement during transfer (CSC NIHSS 8 vs. 16; within-patient median change + 4; *P* = 0.042). Their functional outcome was favorable (discharge mRS median 2, IQR 1–3). In multivariable Firth penalized logistic regression, intravenous thrombolysis was associated with complete recanalization (OR 4.87, 95% CI 1.08–46.25; *P* = 0.039). Moreover, greater neurological improvement showed a consistent direction of association (OR 1.09, 95% CI 1.00–1.18; *P* = 0.059) (Table 2A). Sensitivity analyses using conventional logistic regression and additional adjustment for transfer duration yielded comparable results. Transfer duration was not associated with complete recanalization (OR 1.00, 95% CI 0.98–1.01; *P* = 0.84).

### Extensive infarct progression

Baseline characteristics according to infarct progression are presented in Supplementary Table 1B. At the PSC, patients who subsequently progressed had a near-normal CT ASPECTS (median 9) but a markedly lower CTASI ASPECTS (median 3 vs. 8; *P* < 0.001) and poorer collaterals (median Tan score 1 vs. 2; *P* < 0.001). These patients deteriorated neurologically during transfer (within-patient median NIHSS change − 3; *P* = 0.012) and had poor outcomes (discharge mRS median 6). Greater neurological deterioration (OR 0.83, 95% CI 0.70–0.97; *P* = 0.018) and lower CTASI ASPECTS (OR 0.55, 95% CI 0.34–0.79; *P* = 0.001) were associated with extensive infarct progression, whereas collateral status showed a similar direction of association but did not reach statistical significance (OR 0.33, 95% CI 0.08–1.14; *P* = 0.080) (Table 2B). Sensitivity analyses yielded comparable results: transfer duration was likewise not associated with infarct progression (OR 1.00, 95% CI 0.98–1.01; *P* = 0.78).

### Exploratory analyses

In exploratory analyses, carotid and tandem occlusions had lower odds of complete recanalization than M1 occlusions (OR 0.11, 95% CI 0.001–0.88; *P* = 0.034) and showed a trend toward greater infarct progression (OR 3.26, 95% CI 0.89–12.56; *P* = 0.074). No significant differences were observed between M1 and M2 occlusions, although only seven patients had M2 occlusions. Model diagnostics demonstrated no evidence of relevant multicollinearity (all variance inflation factors < 1.3), and no influential observations materially affected the primary analyses.

## Discussion

In this retrospective cohort of patients with anterior circulation LVO transferred for EVT, clinically meaningful changes during interhospital transfer altered EVT eligibility in approximately one in ten patients. Complete recanalization on repeat CTA occurred in 5% of patients, whereas extensive infarct progression precluding EVT occurred in 4.5%. These findings support structured post-transfer reassessment while suggesting that repeat imaging should be applied selectively rather than routinely.

Our findings extend previous work by demonstrating that neurological evolution during transfer reflects two distinct pathophysiological trajectories. Neurological improvement was associated with complete recanalization on repeat CTA, whereas neurological deterioration was associated with extensive infarct progression on repeat imaging. These observations suggest that changes in neurological status mirror the balance between interval reperfusion and ongoing ischemic injury. By linking neurological evolution with objective imaging findings, our study provides insight into why some transferred patients no longer require EVT whereas others become ineligible because of infarct progression. These findings emphasize the importance of structured neurological reassessment immediately after arrival at the CSC.

Lower baseline CTASI ASPECTS showed the strongest association with extensive infarct progression, indicating that the extent of early ischemic injury is closely related to subsequent infarct evolution [12]. The apparent discrepancy between a near-normal non-contrast CT ASPECTS and a low CTASI ASPECTS on the same hyperacute examination is expected rather than artefactual in patients with a persisting LVO. Non-contrast CT is frequently normal before cytotoxic edema produces frank hypoattenuation, whereas CTASI, weighted toward cerebral blood volume, reveal the extent of critically hypoperfused tissue [13, 14]. In our cohort this low baseline CTASI ASPECTS coexisted with poor collaterals and preceded the subsequent decline in non-contrast-CT ASPECTS, supporting a wider role for CTASI ASPECTS in the hyperacute transfer setting, especially at PSC where CT Perfusion is not comprehensively available [15]. Fast image acquisition and decreased injection flow rates of contrast medium, however, can lead to an overestimation of the infarct size in the CTASI ASPECTS [16]. 

Poorer collateral status was associated with extensive infarct progression on univariable analysis and showed the same direction of effect in the multivariable model, where it was attenuated by the small number of events and its collinearity with early ischemic extent on CTASI ASPECTS. This is concordant with previous studies, which identified collateral status as the principal determinant of ASPECTS decay during interfacility transfer and linked decay to worse functional outcomes [17]. Together these observations indicate that baseline collateral status and CTASI ASPECTS jointly identify the tissue-level substrate for progression during transfer.

The absence of an association between transfer duration and either outcome should not be interpreted as evidence that time is unimportant. Recanalization and infarct progression are time-dependent processes, and because repeat imaging was obtained a median of roughly 2.5 h after thrombolysis we could not determine when recanalization occurred. Our cohort was transferred rapidly within a narrow time window, and with few outcome events statistical power to detect a time effect was limited. Treatment speed therefore remains a central objective; our findings simply indicate that, within this highly selected and rapidly transferred cohort, baseline imaging characteristics were more informative than the transfer interval for identifying patients at risk of a clinically meaningful change.

Intravenous thrombolysis was associated with complete recanalization, consistent with its established role in thrombus dissolution. However, most thrombolyzed patients did not recanalize (103 of 190 patients in the non-recanalized group had received thrombolysis). Thrombolysis is therefore associated with, but far from specific for, interval recanalization and cannot by itself justify repeat imaging; it should be regarded as one input to a combined clinical-and-imaging assessment rather than a stand-alone trigger.

Exploratory analyses further suggested lower odds of complete recanalization and greater infarct progression among carotid and tandem occlusions than M1 occlusions, although these findings require confirmation in larger cohorts.

**Fig. 2:**
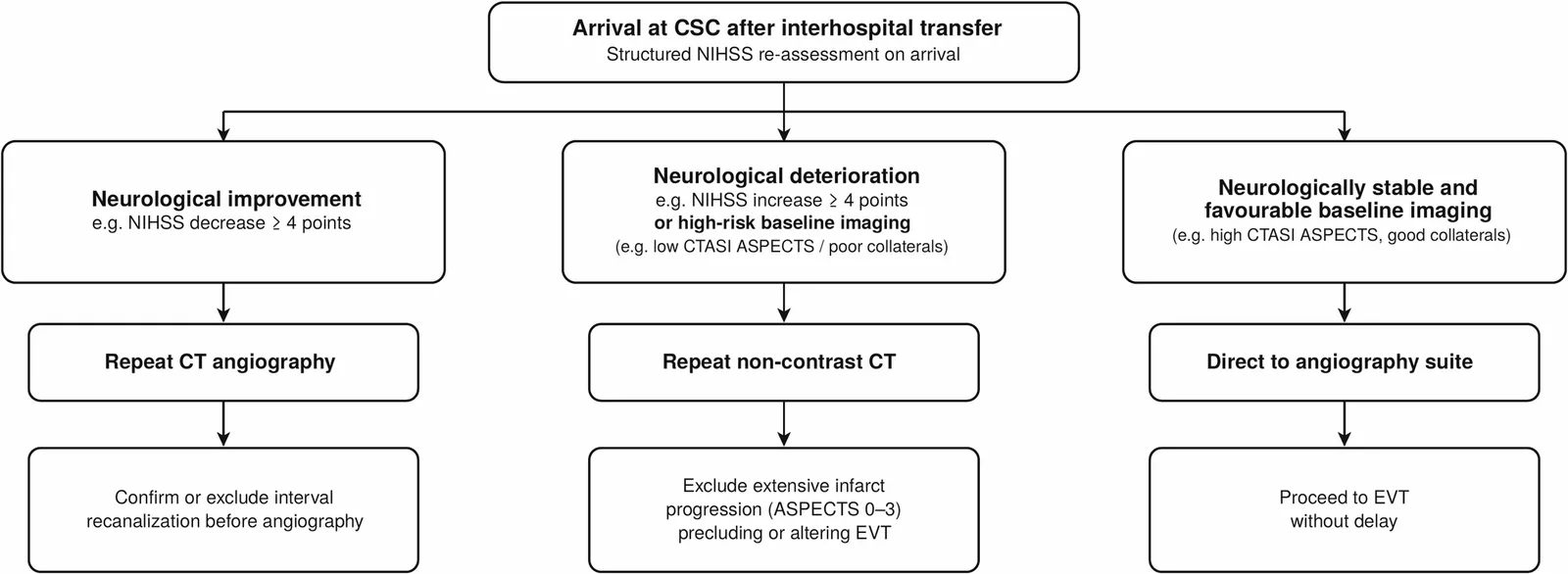
Proposed selective post-transfer reassessment pathway. Neurological change on arrival is the primary trigger; Baseline primary stroke center imaging (CTASI ASPECTS, collateral score) in conjunction with the non-contrast CT is the modifier and refines the classification, especially for clinically stable patients. Proposed pathway derived from the observed associations - to be validated. ASPECTS, Alberta Stroke Program Early CT Score; CT, computed tomography; CTA, computed tomography angiography; CTASI, computed tomography angiography source images; CSC, comprehensive stroke center; EVT, endovascular treatment; NIHSS, National Institutes of Health Stroke Scale

From a clinical perspective, these findings argue against both routine repeat imaging for all transferred patients and direct transfer of every patient to the angiography suite without reassessment. Instead, they support a selective post-transfer assessment strategy integrating neurological reassessment with baseline imaging characteristics. Patients with neurological improvement may warrant evaluation for interval recanalization, whereas neurological deterioration may prompt consideration of infarct progression and repeat imaging. Conversely, patients with stable neurological findings and favorable baseline imaging may be candidates for streamlined workflows directly to the angiosuite upon arrival to the CSC. Whether such a selective approach (Fig. 2) improves efficiency without compromising patient outcomes should be evaluated prospectively.

This study has several limitations. First, its retrospective single stroke network design limits causal inference and generalizability. NIHSS was scored by different trained assessors across network sites, introducing potential inter-rater variability. Collateral status was graded on single-phase CTA, which may underestimate collateral extent relative to multiphase imaging. CTASI ASPECTS is not an established standard-of-care marker and has recognized limitations. Second, the small number of outcome events necessitated Firth penalized logistic regression and restricted the complexity of the multivariable models; consequently, the exploratory analyses of occlusion location should be interpreted cautiously. Third, the study reflects clinical practice between 2016 and 2020, when only patients with an ASPECTS ≥ 5 were transferred for EVT evaluation and intravenous alteplase was the standard thrombolytic agent. Finally, the absence of an association between transfer duration and the primary outcomes should be interpreted within the context of a highly selected cohort undergoing rapid interhospital transfer and should not be interpreted as evidence that treatment delays are clinically unimportant.

Because approximately nine in ten transferred patients showed no change affecting EVT eligibility, routine repeat imaging of all patients is difficult to justify. A structured neurological reassessment on arrival, interpreted together with baseline CTASI ASPECTS and collateral status, identifies the minority most likely to have recanalized or progressed and supports a selective rather than routine repeat-imaging strategy after interhospital transfer.

## Supplementary Information

Supplementary material 1.

## Data Availability

Data availability The datasets used and analyzed during the current study are available from the corresponding author on reasonable request.
