# Supplementary material for: Post-transfer reassessment for endovascular treatment: factors associated with changes in EVT eligibility
Source: Neurol Res Pract. 2026 Oct 9;8(1):83. doi: 10.1186/s42466-026-00539-8 (PMC13651026; doi:10.1186/s42466-026-00539-8)
Supplement: Supplementary file 1 [file 42466_2026_539_MOESM1_ESM.docx]

# **Post-Transfer Reassessment for Endovascular Treatment: Factors Associated with Changes in EVT Eligibility**

## **Supplementary Table 1A.** Baseline characteristics according to complete recanalization

| **Characteristic** | **No complete recanalization (n=190)** | **Complete recanalization (n=10)** | **P value** |
| --- | --- | --- | --- |
| **Demographics** |  |  |  |
| Age (years) | 78 (67–83) | 73 (57–84) | 0.328 |
| Male sex | 83 (43.7) | 5 (50.0) | 0.752 |
| **Medical history** |  |  |  |
| Hypertension | 166/187 (88.8) | 8/9 (88.9) | 1.000 |
| Diabetes mellitus | 77/187 (41.2) | 5/9 (55.6) | 0.495 |
| Atrial fibrillation | 111/187 (59.4) | 5/9 (55.6) | 1.000 |
| Previous stroke | 42/187 (22.5) | 1/9 (11.1) | 0.687 |
| Anticoagulation | 159 (83.7) | 7 (70.0) | 0.379 |
| **Stroke characteristics** |  |  |  |
| Occlusion site | See Supplementary Table 2 | See Supplementary Table 2 | 0.283 |
| Extracranial ICA stenosis/occlusion (>90%) | 38 (20.0) | 1 (10.0) | 0.690 |
| **Clinical variables** |  |  |  |
| NIHSS at referring hospital | 16 (11–20) | 14 (11–17) | 0.199 |
| Intravenous thrombolysis | 103 (54.2) | 9 (90.0) | **0.045** |
| **Imaging** |  |  |  |
| ASPECTS | 9 (8–10) | 10 (10–10) | 0.051 |
| CTA source-image ASPECTS | 7 (6–9) | 10 (8–10) | **0.003** |
| Collateral score | 2 (1–2) | 2 (2–3) | 0.292 |
| **Workflow** |  |  |  |
| Onset-to-imaging (min) | 81 (62–132) | 89 (57–160) | 0.891 |
| Onset-to-IVT (min)* | 112 (89–140) | 145 (103–193) | 0.071 |
| Door-in–door-out time (min) | 109 (86–133) | 104 (81–122) | 0.737 |
| Transfer duration (min)† | 148 (119–174) | 141 (117–159) | 0.551 |
| Door-to-imaging at CSC (min) | 12 (9–17) | 12 (10–19) | 0.897 |
| Onset-to-imaging at CSC (min) | 233 (196–319) | 231 (204–283) | 0.823 |
| **Clinical/imaging evolution** |  |  |  |
| NIHSS at CSC | 16 (10–20) | 8 (5–15) | **0.007** |
| NIHSS change during transfer‡ | 0 (−3 to 3) | 4 (−1 to 11) | **0.042** |
| ASPECTS at CSC | 8 (6–9) | 10 (8–10) | **0.008** |
| CTA source-image ASPECTS at CSC | 7 (5–8) | 10 (8-10) | **<0.001** |
| Collateral score at CSC | 2 (2–2) | 2 (2–3) | 0.075 |

**Supplementary Table 1B**. Baseline characteristics according to extensive infarct progression

| **Characteristic** | **ASPECTS 4–10 (n=191)** | **ASPECTS 0–3 (n=9)** | **P**  **value** |
| --- | --- | --- | --- |
| **Demographics** |  |  |  |
| Age (years) | 77 (67–83) | 77 (67–83) | 0.920 |
| Male sex | 83 (43.5) | 5 (55.6) | 0.511 |
| **Medical history** |  |  |  |
| Hypertension | 168/187 (89.8) | 6/9 (66.7) | 0.066 |
| Diabetes mellitus | 79/187 (42.2) | 3/9 (33.3) | 0.737 |
| Atrial fibrillation | 112/187 (59.9) | 4/9 (88.9) | 0.490 |
| Previous stroke | 43/187 (23.0) | 0/9 (0) | 0.210 |
| Anticoagulation | 159 (83.2) | 7 (70.0) | 0.651 |
| **Stroke characteristics** |  |  |  |
| Occlusion site | See Supplementary Table 2 | See Supplementary Table 2 | 0.279 |
| Extracranial ICA stenosis/occlusion (>90%) | 36 (18.8) | 3 (33.3) | 0.381 |
| **Clinical variables** |  |  |  |
| NIHSS at referring hospital | 15 (11–19) | 19 (17–20) | 0.057 |
| Intravenous thrombolysis | 105 (55.0) | 7 (77.8) | 0.304 |
| **Imaging** |  |  |  |
| ASPECTS | 9 (8–10) | 9 (8–10) | 0.882 |
| CTA source-image ASPECTS | 8 (6–9) | 3 (2–5) | **<0.001** |
| Collateral score | 2 (2–3) | 1 (1–1) | **<0.001** |
| **Workflow** |  |  |  |
| Onset-to-imaging (min) | 81 (61–138) | 75 (67–95) | 0.780 |
| Onset-to-IVT (min)* | 115 (90–145) | 105 (95–135) | 0.824 |
| Door-in–door-out time (min) | 107 (85–130) | 118 (94–146) | 0.302 |
| Transfer duration (min)† | 147 (119–173) | 150 (121–187) | 0.557 |
| Door-to-imaging at CSC (min) | 12 (9–17) | 13 (12–16) | 0.425 |
| Onset-to-imaging at CSC (min) | 235 (195–319) | 229 (211–283) | 0.861 |
| **Clinical/imaging evolution** |  |  |  |
| NIHSS at CSC | 16 (9–20) | 22 (18–32) | **0.002** |
| NIHSS change during transfer‡ | 0 (−3 to 4) | −3 (−11 to −1) | **0.012** |
| ASPECTS at CSC | 8 (7–9) | 3 (2–3) | **<0.001** |
| CTA source-image ASPECTS at CSC | 7 (5–8) | 2 (1-3) | **<0.001** |
| Collateral score at CSC | 2 (2–2) | 1 (1–2) | **0.007** |

Data are presented as median (interquartile range) or n (%). Variable-specific denominators indicate missing data.

*Among patients receiving intravenous thrombolysis.

†Door-out at the referring hospital to arrival at the comprehensive stroke center. ‡Calculated as NIHSS at the referring hospital minus NIHSS at the comprehensive stroke center; positive values indicate neurological improvement and negative values indicate neurological deterioration.

**Supplementary Table 2**. Distribution of stroke etiology and occlusion location according to the primary outcomes.

| **Occlusion location** | **Overall** | **Complete**  **recanalization** | **Extensive infarct**  **progression** |
| --- | --- | --- | --- |
| Carotid/tandem | 55 | 1 (1.8%) | 3 (5.5%) |
| M1 | 138 | 8 (5.8%) | 5 (3.6%) |
| M2 | 7 | 1 (14.3%) | 1 (14.3%) |
